# Method for a new risk assessment of urban inundation: G-DEMATEL–AHP

**DOI:** 10.1016/j.mex.2023.101997

**Published:** 2023-01-10

**Authors:** Qian Zheng

**Affiliations:** a Department of Civil and Environmental Engineering, College of Engineering, Shantou University, Shantou, Guangdong 515063, China; b Discipline of Civil and Infrastructure Engineering, School of Engineering, Royal Melbourne Institute of Technology (RMIT), Victoria 3001, Australia

**Keywords:** Decision-making trial and evaluation laboratory, Analytical hierarchical process, Urban inundation, Risk assessment, Method of integrating grey theory, DEMATEL, and AHP for risk assessment

## Abstract

Urban flooding disasters caused by frequent heavy rainfall threaten the safety of residents and socioeconomic development. Thus, risk evaluation of urban inundation is a vital component of sustainable urban development. Analytical hierarchical process (AHP) and decision-making trial and evaluation laboratory (DEMATEL) are common methods applied to flood risk evaluation. The former focuses on the relationship among different layers, while the latter reflects the relationship of factors in one layer. Moreover, the ambiguity in the decision-making process can be reduced by the uncertainty system. Therefore, the proposed method integrates grey theory and utilises the characteristics of DEMATEL to establish the AHP comparison matrix, particularly when the scores given by experts’ questionnaires are scattered. The method was applied to the risk assessment of urban inundation in Zhengzhou and verified by its flooding disaster sites that occurred during the 20 July storm in 2021 [1].

• Developed approach merges grey theory, DEMATEL, and AHP methods;
• Application of G-DEMATEL-AHP method in risk assessment of urban inundation;
• The proposed method can increase the reliability of risk assessment results.

## Graphical Abstract

**Figure:**
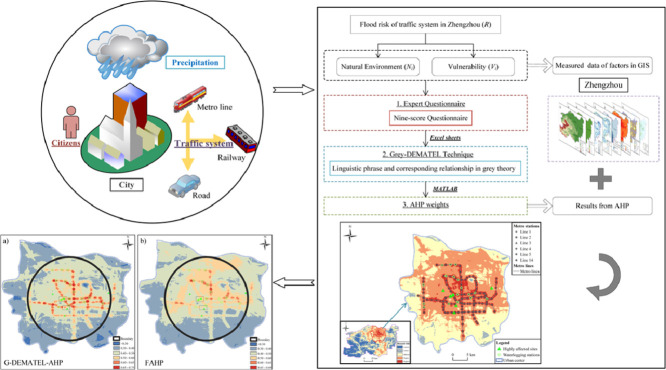

Specifications Table

**Table:** 

Subject area	Engineering
More specific subject area	Urban inundation evaluation
Method	Method of integrating grey theory, DEMATEL, and AHP for risk assessment
Name and reference of the original method	Saaty, T.L., (1977). A scaling method for priorities in hierarchical structures. *Journal of Mathematical Psychology*, 15, 234–281, doi: https://doi.org/10.1016/0022-2496(77)90033-5 [8].
Resource availability	DOI: https://doi.org/10.1016/j.scs.2022.104138

## Data and methods

### Data from expert questionnaire survey

The expert survey used a nine-score questionnaire [2]. The score (*x_i_, x_j_*) gained by considering the importance degree of the *n_th_* factor in its layer ranged from 1 to 9. The relative importance *x_ij,k_* between factors in the same layer was calculated using the following equations:

(1) $$x_{ij,\;\;k}=\theta_{k}\frac{x_{i,k}}{x_{j,k}},\;x_{i=j,k}=0$$

where *x_i,k_* and *x_j,k_* are directly obtained from the questionnaire, *x_i,k_, x_j,k_* ∈[1, 9], *k* is the *k_th_* expert, and *θ_k_* is the relative importance coefficient of the *k_th_* expert, which can be obtained as follows:

(2) $$\theta_{k}=\frac{\text{max}\left(x_{i,k}\right)}{9},\;i=1,\;2,\ldots ,\;n$$

### Method details

**Step 1:** According to the data from the expert questionnaire survey and Eq. (1)–(2), the direct influential relation matrix *C_k_^’^* from the *k_th_* expert can be expressed using the following equation:

(3) $${C^{\prime }}_{k}={\left[\begin{array}{cccc} 0 & x_{12,k} & \ldots & x_{1n,k}\\ x_{21,k} & \ddots & \ldots & x_{2n,k}\\ \vdots & \ldots & 0 & \vdots \\ x_{n1,k}\; & x_{n2,k} & \ldots & 0 \end{array}\right]}_{n\times n}$$

**Step 2:** The *k_th_* direct influential relation matrix was normalised. That of each expert can be normalised as *C_k_* using the following equation:

(4) $$C_{k}=\frac{1}{\max\left(x_{ij,k}\right)}\times {C^{\prime }}_{k}={\left[\begin{array}{cccc} 0 & c_{12,k} & \ldots & c_{1n,k}\\ c_{21,k} & \ddots & \ldots & c_{2n,k}\\ \vdots & \ldots & 0 & \vdots \\ c_{n1,k}\; & c_{n2,k} & \ldots & 0 \end{array}\right]}_{n\times n}$$

**Step 3:** Fuzzification using Grey theory. The value of *c_ij,k_* can be fuzzified into interval grey numbers according to Grey theory [3]. The corresponding relationship between the interval grey number and its operating algorithm are presented in Table 4 and Eq. (3)–(5) in a companion study [1]. Therefore, the elements of matrix *C_k_* can be transformed into the grey interval number as follows [4]:

$$C_{k}\Rightarrow \left[C_{k}^{down},C_{k}^{up}\right]$$

(5) $$\begin{array}{c} \left[C_{k}^{\mathrm{down}},C_{k}^{\mathrm{up}}\right]={\left[\begin{array}{cccc} 0 & c_{12,k}^{\;\mathrm{down}} & \cdots & c_{1n,k}^{\;\mathrm{down}}\\ c_{21,k}^{\;\mathrm{down}} & \ddots & \cdots & c_{2n,k}^{\;\mathrm{down}}\\ \vdots & \cdots & 0 & \vdots \\ c_{n1,k}^{\;\mathrm{down}} & c_{n2,k}^{\;\mathrm{down}} & \cdots & 0 \end{array},\begin{array}{cccc} 0 & c_{12,k}^{\;\mathrm{up}} & \cdots & c_{1n,k}^{\;\mathrm{up}}\\ c_{21,k}^{\;\mathrm{up}} & \ddots & \cdots & c_{2n,k}^{\;\mathrm{up}}\\ \vdots & \cdots & 0 & \vdots \\ c_{n1,k}^{\;\mathrm{up}} & c_{n2,k}^{\;\mathrm{up}} & \cdots & 0 \end{array}\right]}_{n\times n} \end{array}$$

**Step 4:** Averaging the comprehensive influential relation matrix (N), which is composed of the upper limit matrix (*N^up^*) and lower limit matrix (*N^down^*), consisting of the average value *c_ij_* of the upper and lower bounds from all experts’ influence scores (*c_ij,k_*), respectively. Eq. (6) shows the comprehensive influential relation matrix using grey theory.

(6) $$\begin{array}{ccc} N=\left[N^{down},N^{up}\right] & = & {\left[\begin{array}{cccc} 0 & {\bar{c}}_{12}^{\;down} & \cdots & {\bar{c}}_{1n}^{\;down}\\ {\bar{c}}_{21}^{\;down} & \ddots & \cdots & {\bar{c}}_{2n}^{\;down}\\ \vdots & \cdots & 0 & \vdots \\ {\bar{c}}_{n1}^{\;down} & {\bar{c}}_{n2}^{\;down} & \cdots & 0 \end{array},\begin{array}{cccc} 0 & {\bar{c}}_{12}^{\;up} & \cdots & {\bar{c}}_{1n}^{\;up}\\ {\bar{c}}_{21}^{\;up} & \ddots & \cdots & {\bar{c}}_{2n}^{\;up}\\ \vdots & \cdots & 0 & \vdots \\ {\bar{c}}_{n1}^{\;up} & {\bar{c}}_{n2}^{\;up} & \cdots & 0 \end{array}\right]}_{n\times n}\\ & = & {\left(\begin{array}{cccc} \left[0,0\right] & \left[{\bar{c}}_{12}^{\;down},{\bar{c}}_{12}^{\;up}\right] & \cdots & \left[{\bar{c}}_{1n}^{\;down},{\bar{c}}_{1n}^{\;up}\right]\\ \left[{\bar{c}}_{21}^{\;down},{\bar{c}}_{21}^{\;up}\right] & \ddots & \cdots & \left[{\bar{c}}_{2n}^{\;down},{\bar{c}}_{2n}^{\;up}\right]\\ \vdots & \cdots & \left[0,0\right] & \vdots \\ \left[{\bar{c}}_{n1}^{\;down},{\bar{c}}_{n1}^{\;up}\right] & \left[{\bar{c}}_{n2}^{\;down},{\bar{c}}_{n2}^{\;up}\right] & \ldots & \left[0,0\right] \end{array}\right)}_{n\times n} \end{array}$$

where ${\bar{c}}_{ij}^{\;down}=\frac{\sum_{k=1}^{K}c_{ij,k}^{down}}{K}$, ${\bar{c}}_{ij}^{\;up}=\frac{\sum_{k=1}^{K}c_{ij,k}^{up}}{K}$, (7) where *K* is the total number of experts.

**Step 5:** Whitenization of Grey interval number. Furthermore, to whiten the comprehensive influential relation matrix [*N^down^, N^up^*], the method selects the crisp value from each grey interval using Eq. (8)–(11) [5].

(8) $${\bar{c}}_{ij,nor}^{\;up}=\frac{{\bar{c}}_{ij}^{\;up}-\min_{j}\left({\bar{c}}_{ij}^{\;up}\right)}{\Delta_{min}^{max}}$$

(9) $${\bar{c}}_{ij,nor}^{\;down}=\frac{{\bar{c}}_{ij}^{\;down}-\min_{j}\left({\bar{c}}_{ij}^{\;down}\right)}{\Delta_{min}^{max}}$$

(10) $$\Delta_{min}^{max}=\max_{j}{\bar{c}}_{ij}^{\;up}-\min_{j}{\bar{c}}_{ij}^{\;down}$$

(11) $$s_{ij}=\min_{j}{\bar{c}}_{ij}^{\;down}+\frac{{\bar{c}}_{ij,nor}^{\;down}\left(1-{\bar{c}}_{ij,nor}^{\;down}\right)+\left({\bar{c}}_{ij,nor}^{\;up}\times {\bar{c}}_{ij,nor}^{\;up}\right)}{1-{\bar{c}}_{ij,nor}^{\;down}+{\bar{c}}_{ij,nor}^{\;up}}$$

As the minimum value of the upper influential relation matrix must be zero, Eq. (8)–(11) can be simplified as Eq. (15)–(17), respectively.

(12) $${\bar{c}}_{ij,nor}^{\;up}=\frac{{\bar{c}}_{ij}^{\;up}}{\max_{j}{\bar{c}}_{ij}^{\;up}}$$

(13) $${\bar{c}}_{ij,nor}^{\;down}=\frac{{\bar{c}}_{ij}^{\;down}}{\max_{j}{\bar{c}}_{ij}^{\;up}}$$

(14) $$\Delta_{min}^{max}=\max_{j}{\bar{c}}_{ij}^{\;up}$$

Finally, crisp values (*s_ij_*) were calculated using Eq. (15):

(15) $$s_{ij}=\frac{{\bar{c}}_{ij}^{\;down}-{\bar{c}}_{ij}^{\;down}\times {\bar{c}}_{ij,nor}^{\;down}+{\bar{c}}_{ij}^{\;up}\times {\bar{c}}_{ij,nor}^{\;up}}{\max_{j}{\bar{c}}_{ij}^{\;up}-{\bar{c}}_{ij,nor}^{\;down}+{\bar{c}}_{ij,nor}^{\;up}}$$

**Step 6:** Degree of influence of DEMATEL. Based on the results obtained using Eq. (15), *s_ij_* can compose a new crisp influential matrix (*S*), which can be expressed as Eq. (16). Then, the total relation matrix (*T*) can be calculated using Eq. (17). The influential degree (*D_i_*) and influenced degree (*R_i_*) of the *i_th_* factor can be obtained from matrix *T*, as expressed in Eq. (19).

(16) $$S={\left[\begin{array}{cccc} 0 & s_{12} & \ldots & s_{1n}\\ s_{21} & \ddots & \ldots & s_{2n}\\ \vdots & \ldots & 0 & \vdots \\ s_{n1}\; & s_{n2} & \ldots & 0 \end{array}\right]}_{n\times n}$$

(17) $$T=S_{nor}{\left(E-S_{nor}\right)}^{-1}$$

Where

(18) $$S_{nor}=\frac{1}{\max_{1\leq i\leq n}\sum_{j}s_{ij}}\times S,\;i,j=1,\ldots ,\;n.$$

(19) $$D_{i}=\sum_{j=1}^{n}T_{ij},\;R_{i}=\sum_{i=1}^{n}T_{ij}$$

**Step 7:** Weights using AHP. The comparison matrix (*A*) of the AHP was established by pairwise comparison of *D_i_*. Detailed information on the AHP calculation procedure can be found in a companion article [1]. The final weight (*ѡ_i_*) for each factor was obtained from this step.

## Application of the method

The traditional AHP approach has the following limitations: (i) the judgements from experts have uncertainty [6], and (ii) choosing suitable scores from experts’ scattered judgements is difficult [7]. Additionally, both AHP and DEMATEL require a questionnaire survey from experts. However, the typical pairwise comparison questionnaires of these two methods are different [8], [9], [10], [11], which makes the procedure of questionnaire evaluation repetitive and verbose [12], [13], [14], [15], [16], [17]. Therefore, the proposed method first simplified the expert questionnaire steps of DEMATEL and AHP through a nine-score questionnaire, and then combined these two MCDM methods with Grey theory to consider all experts’ judgements and calculate the weights. Eq. (20) lists the index layer *N_i_* comparison matrix (*A_Ni_*) for risk assessment of flooding disasters. The details of the risk assessment frame are indicated in a companion article [1].

(20) $$A_{N_{i}}={\left(a_{ij}\right)}_{n\times n}={\left[\begin{array}{cc} \begin{array}{ccc} 1.00 & 1.05 & 1.13\\ 0.95 & 1.00 & 1.07\\ 0.89 & 0.93 & 1.00 \end{array} & \begin{array}{ccc} 1.29 & 0.74 & 0.78\\ 1.23 & 0.70 & 0.74\\ 1.15 & 0.66 & 0.69 \end{array}\\ \begin{array}{ccc} 0.77 & 0.81 & 0.87\\ 1.35 & 1.42 & 1.52\\ 1.29 & 1.35 & 1.45 \end{array} & \begin{array}{ccc} 1.00 & 0.57 & 0.60\\ 1.75 & 1.00 & 1.05\\ 1.66 & 0.95 & 1.00 \end{array} \end{array}\right]}_{6\times 6}$$

Based on this method, the consistency ratio (*CR*) of the *N_i_* comparison matrix can be checked, which should be less than 0.1. Meanwhile, the weight (*w_i_*) of each factor belonging to the index layer *N_i_* can be computed using MATLAB software based on Eq. (7)–(9) in a companion article [1]. Therefore, the weights of the factors in the *N_i_* layer were calculated as *w_i_* = (0.1600, 0.1523, 0.1420, 0.1236, 0.2163, and 0.2058).

## Computational tools

Excel sheets (Excel file: Excel for_MethodsX-G-DEMATEL-AHP) and MATLAB software were used to calculate the flooding disaster case in Zhengzhou for further illustration. The Excel file included five sheets: raw data, layer_Id, layer_N_i_, layer_V_i_, and the result. Based on the assessment frame in the companion article [1], the Excel sheet can calculate the influential degree (*D_i_*) of the factors in each layer. MATLAB software was used to solve the AHP comparison matrix composed of *D_i_*. Finally, the weight of each factor in each layer is shown in the result sheet. Therefore, the detailed steps for using the calculation tool are as follows: first, the scores from the nine-score questionnaire were collected and input into the corresponding area in the raw data sheet. Second, the raw data was divided into different sheets (Layer_Id, Layer_N_i_, and Layer_V_i_) and the *D_i_* value of each factor was calculated to establish the AHP comparison matrix in the navy-blue area. Third, MATLAB software was used to solve the comparison matrices and gain the weights (*ѡ_i_*) of the factors in each index layer. Finally, all values of the weights in the sheet result were input to calculate the comprehensive weight (*ѡ_i’_*) of each factor.

## Method validation

To test the advantages of the proposed G-DEMTAL-AHP method, the weights of the factors with the corresponding actual data from Zhengzhou were mapped using GIS software. The distribution of risk levels could be obtained from this step, which was compared with the waterlogging sites that occurred during the Zhengzhou flooding disaster on 20 July 2021. Detailed information on dividing risk levels and relative analyses can be found in Section 4.2. of a companion article [1]. Moreover, the existing TFN-AHP method was used to evaluate the flooding risk level in the Zhengzhou case. The results of the weights and risk distribution of the two methods are presented in details in the companion article [1].

## Declaration of Competing Interest

The authors declare that they have no known competing financial interests or personal relationships that could have appeared to influence the work reported in this paper.

## Data Availability

Data will be made available on request.

## Appendix Supplementary materials

Image, application 1

## References

[1] Zheng Q., Shen S.L., Zhou A., Lyu H.M. (2022). Inundation risk assessment based on G-DEMATEL-AHP and its application to Zhengzhou flooding disaster. Sustainable Cities and Society.

[2] Lyu H.M., Sun W.J., Shen S.L., Zhou A. (2020). Risk assessment using a new consulting process in fuzzy AHP. Journal of Construction Engineering and Management.

[3] Deng J.L. (1989). Introduction to grey system theory. The J. Grey Syst..

[4] Dou Y., Sarkis J. (2013). A multiple stakeholder perspective on barriers to implementing China RoHS regulations. Resources, Conserv. Recycling.

[5] Opricovic S., Tzeng G.H. (2003). Defuzzification within a multicriteria decision model. Int. J. Uncertainty, Fuzziness and Knowledge-Based Syst..

[6] Zheng Q., Lyu H.M., Zhou A., Shen S.L. (2021). Risk assessment of geohazards along Cheng-Kun railway using fuzzy AHP incorporated into GIS. Geomatics, Natural Hazards and Risk.

[7] Lyu H.M., Zhou W.H., Shen S.L., Zhou A. (2020). Inundation risk assessment of metro system using AHP and TFN-AHP in Shenzhen. Sustainable Cities and Society.

[8] Saaty T.L. (1977). A scaling method for priorities in hierarchical structures. J. Mathemat. Psychol..

[9] Wu W.W., Lee Y.T. (2007). Developing global managers’ competencies using the fuzzy DEMATEL method. Expert Syst. Appl..

[10] Chen Y.L., Shen S.L., Zhou A. (2022). Assessment of red tide risk by integrating CRITIC weight method, TOPSIS-ASSETS method, and Monte Carlo simulation. Environ. Pollut..

[11] Yan T., Shen S.L., Zhou A. (2022). Indices and models of surface water quality assessment: review and perspectives. Environ. Pollut..

[12] Shen S.L., Zhang N., Zhou A., Yin Z.Y. (2022). Enhancement of neural networks with an alternative activation function tanhLU. Expert Syst. Appl..

[13] Shen S.L., Lin S.S., Zhou A. (2023). Comprehensive risk status evaluation of excavation system based on entropy-cloud model. Reliability Eng. Syst. Safety.

[14] Lyu H.M., Shen S.L., Zhou A., Yin Z.Y. (2022). Assessment of safety status of shield tunnelling using operational parameters with enhanced SPA. Tunnel. Underground Space Technol..

[15] Lin S.S., Shen S.L., Zhou A., Xu Y.S. (2021). Novel model for risk identification during karst excavation. Reliability Eng. Syst. Safety.

[16] Lin S.S., Zhang N., Zhou A., Shen S.L. (2022). Risk evaluation of excavation based on fuzzy decision-making model. Automation in Construction.

[17] Lyu H.M., Shen S.L., Zhou A. (2021). The development of IFN-SPA: a new risk assessment method of urban water quality and its application in Shanghai. J. Clean. Product..

